# Rare Case of Vallecular Hemangioma With Complete Regression in an Adult Patient

**DOI:** 10.1002/lary.70128

**Published:** 2025-09-12

**Authors:** Guilherme Reimann Agne, Gustavo Nunes Bento, Marcelo Belli, Camila Batista Rossi, Luciana Depiere Lanzarin, Daniel Cury Ogata, Renan Bezerra Lira, Leandro Luongo Matos, Luiz Paulo Kowalski

**Affiliations:** ^1^ Department of Surgery PESCOP Itajaí Santa Catarina Brazil; ^2^ Department of Research PESCOP Itajaí Santa Catarina Brazil; ^3^ Department of Pathology Infolaudo Laboratory Itajaí Santa Catarina Brazil; ^4^ Department of Head and Neck Surgery and Otorhinolaryngology A.C. Camargo Cancer Center São Paulo São Paulo Brazil; ^5^ Department of Head and Neck Surgery Universidade de São Paulo Medical School São Paulo São Paulo Brazil; ^6^ Departament of Surgery Surgery, Hospital Albert Einstein São Paulo São Paulo Brazil

**Keywords:** case report, head and neck hemangioma, hemangioma, regression, vallecular hemangioma

## Abstract

A rare case of spontaneous regression of a pedunculated vallecular hemangioma in a 55‐year‐old male is presented. Following an incisional biopsy, the lesion regressed completely within three weeks, confirmed by imaging and direct laryngoscopy. This case highlights an unusual clinical outcome and raises questions about the mechanisms underlying hemangioma regression in adults.

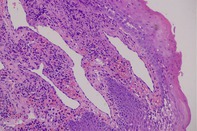

## 1 Introduction

Hemangioma is the most prevalent congenital vascular anomaly. It is characterized by the irregular proliferation of vascular endothelial cells, with 70% of cases identified at birth and 85% occurring within the first year of life. The incidence of hemangiomas is approximately 2.5% in newborns and is considered less common in adults [1].

The most frequently affected locations include the skin, subcutaneous tissue, tongue, nasal mucosa, oral cavity, larynx, and salivary glands. The pharynx represents a rare site for hemangiomas, with only a few cases documented in the literature. Common symptoms of pharyngeal hemangiomas include globus sensation, dysphagia, bleeding, and vocal changes. Diagnosis typically employs nasofibrolaryngoscopy and cross‐sectional imaging techniques [2].

Unlike in pediatric patients, in whom the natural history of hemangiomas often involves spontaneous remission or management primarily involving beta‐blockers, the treatment of pharyngeal hemangiomas in adults usually necessitates surgical intervention [2]. Complete regression of head and neck hemangiomas in adults has been documented in a single reported case of nasal hemangioma following partial resection [3]. Furthermore, spontaneous regression in malignant tumors, while rare, is generally associated with tumors exhibiting high immunological activity [3].

To our knowledge, this report represents the second documented case of vallecular hemangioma and the first instance of spontaneous regression following a small incisional biopsy.

All procedures followed ethical standards and the Declaration of Helsinki. This case report adheres to the CARE checklist guidelines for consistency and quality. Written informed consent was obtained from the patient for publication, including associated images.

## 2 Case Report

**FIGURE 1:**
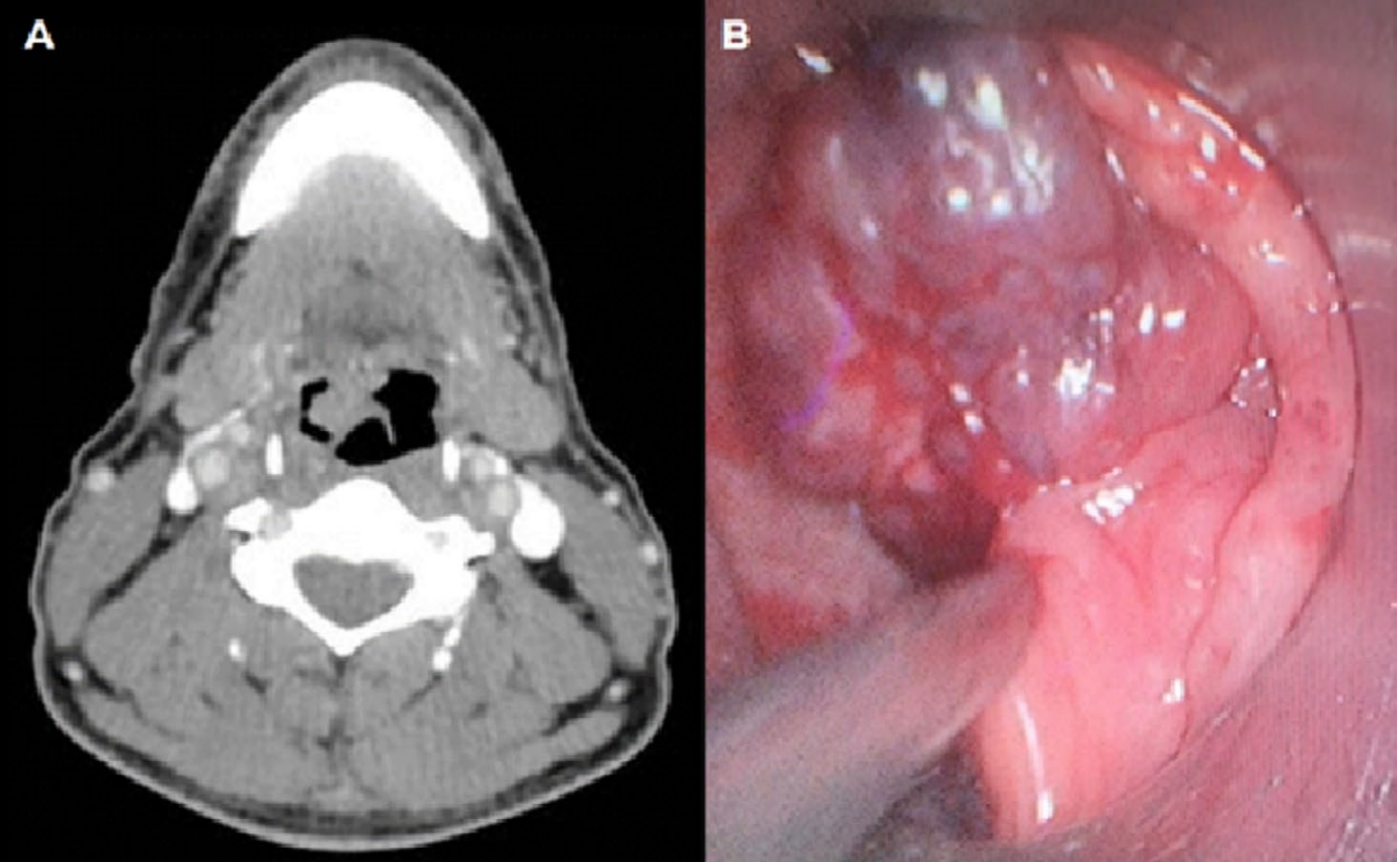
Computed tomography (A) and direct laryngoscopy (B) at the time of incisional biopsy, demonstrating a vallecular lesion suggestive of hemangioma.

A 55‐year‐old male with no significant past medical history presented with a globus sensation and persistent throat inflammation that had been ongoing for several months. During an initial evaluation by an otolaryngologist (ENT specialist), a lesion of the vallecula was identified, leading to a subsequent referral to a head and neck surgeon. A computed tomography (CT) scan revealed a vallecular lesion in close contact with the anterior surface of the epiglottis and the median glossoepiglottic fold, displaying homogeneous enhancement following intravenous contrast administration (Figure 1A).

**FIGURE 2:**
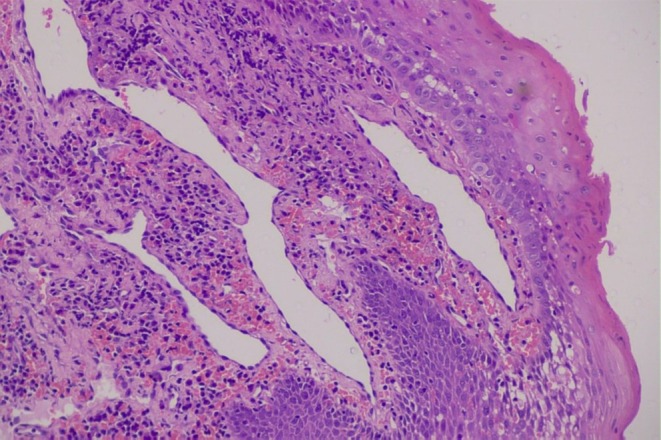
Hematoxylin and eosin‐stained slide showing dilated vascular spaces within the subepithelial connective tissue (magnification, 20×).

Nasofibrolaryngoscopy demonstrated a hypervascular, pedunculated lesion that exhibited minimal mobility within the vallecula. To aid in oral exposure and further characterize the lesion for possible robotic transoral resection (TORS), direct laryngoscopy was performed under general anesthesia. Due to the rarity of vallecular hemangiomas, an incisional biopsy was conducted to document the lesion and exclude malignancy (Figure 1B). A 2 mm tissue fragment was excised, resulting in minimal and easily controllable bleeding. The histopathological analysis revealed dilated vascular spaces within the subepithelial connective tissue, with no evidence of erosion of the overlying stratified squamous epithelium. These vascular spaces were lined by flattened endothelial cells without cytological atypia (Figure 2 and Supporting Information S1). Immunohistochemical staining for CD34 confirmed the vascular origin of the endothelial lining (see Figure S2). Among malignant vascular tumors, angiosarcoma was excluded based on the absence of cellular atypia, and Kaposi's sarcoma was ruled out due to the lack of HHV‐8 immunoexpression (see Figure S3). The diagnosis of hemangioma was confirmed by clinical, radiological, and histopathological findings (see Figure S4).

**FIGURE 3:**
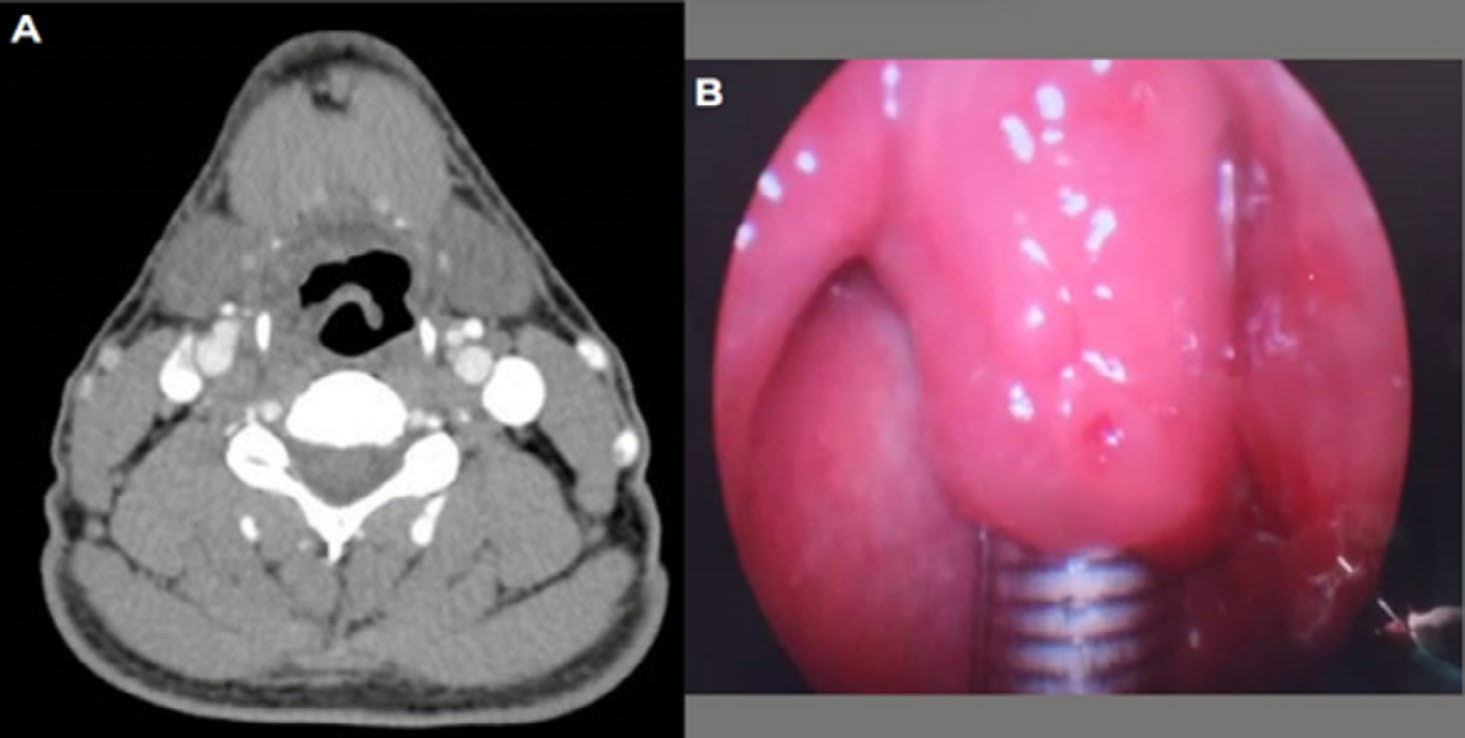
Follow‐up CT scans (A) and direct laryngoscopy (B), demonstrating absence of lesion without any curative intervention.

Three weeks later, the patient returned to the surgical center for the planned TORS procedure. No additional imaging evaluation was performed prior to surgery. During the operation, the inspection with retractors and endoscope revealed no lesion or residual scarring. A CT scan performed during hospitalization confirmed complete remission of the lesion (Figure 3). The patient has remained free of recurrence for 6 months.

## 3 Discussion

Pharyngeal hemangiomas are exceedingly rare entities. Lechien et al. reported a case located in the oropharynx and conducted a review of all cases published in English literature, identifying a total of 12 instances [2]. Among these patients, 45% were male, and the mean age was 45 years. Of the reported cases, 10 involved the hypopharynx, and none were documented in the vallecula. The most common presentation of pharyngeal hemangiomas was flat masses or spots; pedunculated types, while rare, were the only variant documented to date. This type of presentation, akin to our case, is noteworthy as it tends to cause symptoms primarily due to the volume of the lesion, which may render it more amenable to surgical treatment [2].

The sole previously documented case of a hemangioma originating from the vallecula was published in 2020, wherein the lesion caused airway obstruction, necessitating a hybrid technique involving direct laryngoscopy‐assisted fiberscope‐guided intubation prior to resection [4].

In adults, hemangiomas often exhibit persistence over time and typically do not respond favorably to pharmacologic treatment. Given the hypervascular nature of these lesions, surgical intervention is usually the primary treatment option. Nevertheless, alternative strategies, including embolization or active surveillance, may be considered depending on the lesion's size, functional implications, and specific surgical risks. According to Lechien et al., surgical intervention was performed in 82% of cases involving pharyngeal hemangiomas, with laser resection identified as the most commonly employed technique. This method effectively integrates excision and photocoagulation and was first described for hypopharyngeal hemangiomas [2].

In the current case, TORS was planned, a technique successfully utilized in previous laryngeal hemangioma resections. Adequate surgical exposure is critical for TORS, thus necessitating an assessment while the patient was under anesthesia in the operating room. While the decision to perform a biopsy may be considered controversial, it was undertaken in this instance to document the rare lesion. Furthermore, our intention was to exclude malignant pathology and any other differential diagnoses, such as submucosal hematoma. However, performing the biopsy allowed us to confirm the diagnosis we had suspected at that time.

Understanding the mechanisms underlying spontaneous tumor regression remains complex. Lee et al. reported a case of a nasal hemangioma where surgery was halted due to significant hemorrhage, with embolization planned subsequently. The initial material obtained confirmed the diagnosis of lobular capillary hemangioma. The patient opted against further procedures, and complete remission was observed after two years of follow‐up; the authors attributed this tumor regression to decreased vascular endothelial growth factor levels, similar to what is noted in postpartum hemangiomas [3]. Similarly, instances of spontaneous regression have been noted in juvenile nasoangiofibroma, a benign but highly vascular tumor, likely linked to fluctuations in androgenic hormones [5].

Spontaneous regression of malignant tumors, though also rare, is well‐documented and often connected to strong immunogenic responses. Certain malignancies, including renal cell carcinoma, neuroblastoma, malignant melanoma, hepatocellular carcinoma, and choriocarcinoma, are particularly recognized for their high immune activity and propensity for spontaneous regression.

In our case, tumor regression was observed three weeks post‐biopsy. The potential underlying mechanisms may include thrombosis of the hemangioma's feeding vessel, resulting in necrosis, along with possible immunological responses or infection. It is plausible that a combination of these factors contributed to the observed outcome.

## 4 Conclusion

This case report details a pedunculated hemangioma of the vallecula, underscoring its unusual presentation and anatomical location. Notably, the complete regression of the lesion following a small biopsy sheds light on the poorly understood mechanisms of tumor regression.

## Conflicts of Interest

The authors declare no conflicts of interest.

## Supporting information


**Figure S1:** Hematoxylin and eosin (HE) staining (magnification, 40×).

**Figure S2:** Immunohistochemical staining for CD34 (magnification, 20×).

**Figure S3:** Immunohistochemical staining for HHV8 (magnification, 40×).

**Figure S4:** Final pathology report confirming the diagnosis of vallecular hemangioma.
