# Supplementary figures and images for: Rare Case of Vallecular Hemangioma With Complete Regression in an Adult Patient
Source: Laryngoscope. 2025 Sep 12;136(2):794–7. doi: 10.1002/lary.70128 (PMC12793954; doi:10.1002/lary.70128)

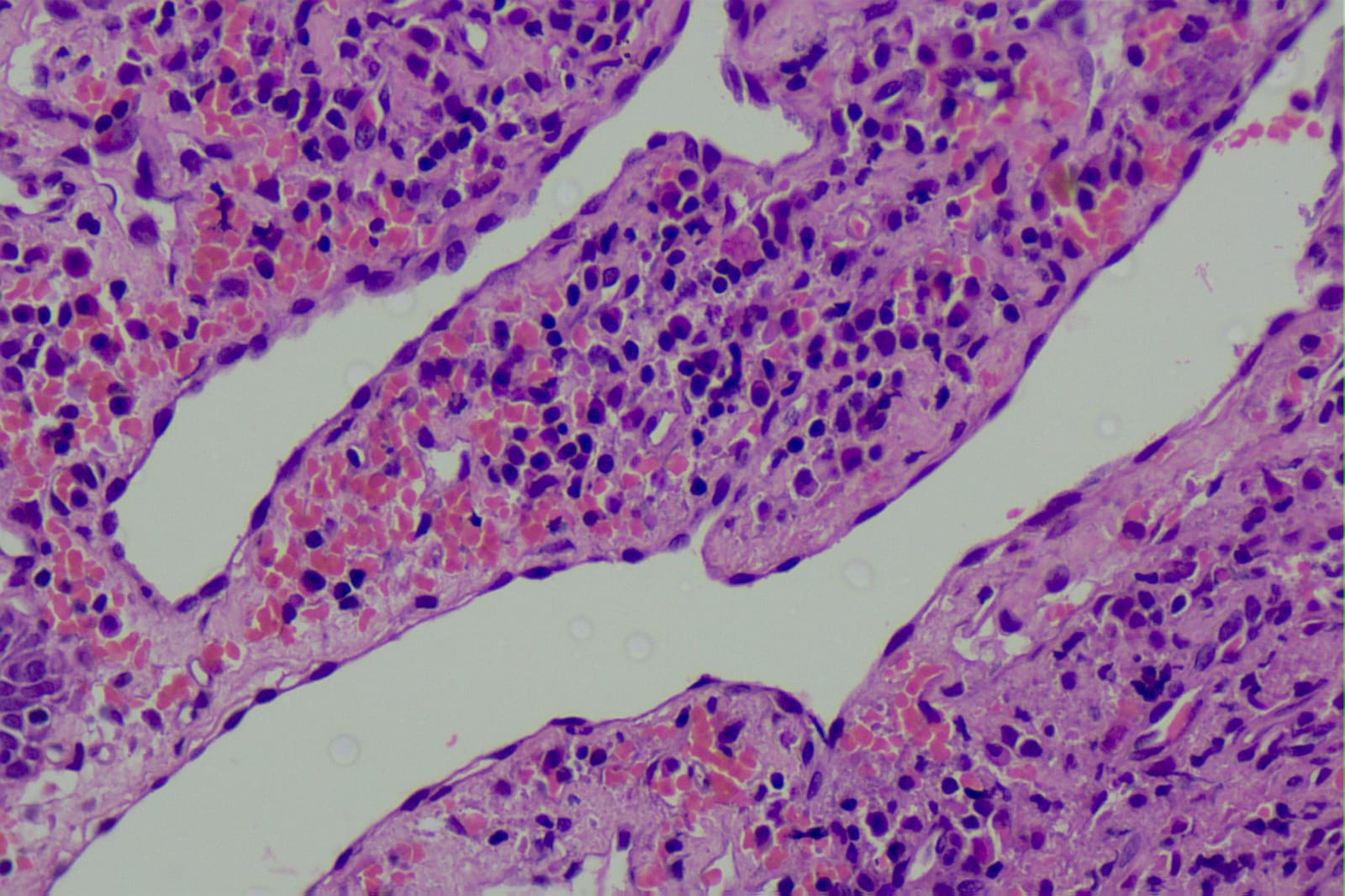

Supplement: Supplementary file 1 — Figure S1: Hematoxylin and eosin (HE) staining (magnification, 40×). [file LARY-136-794-s001.jpg]

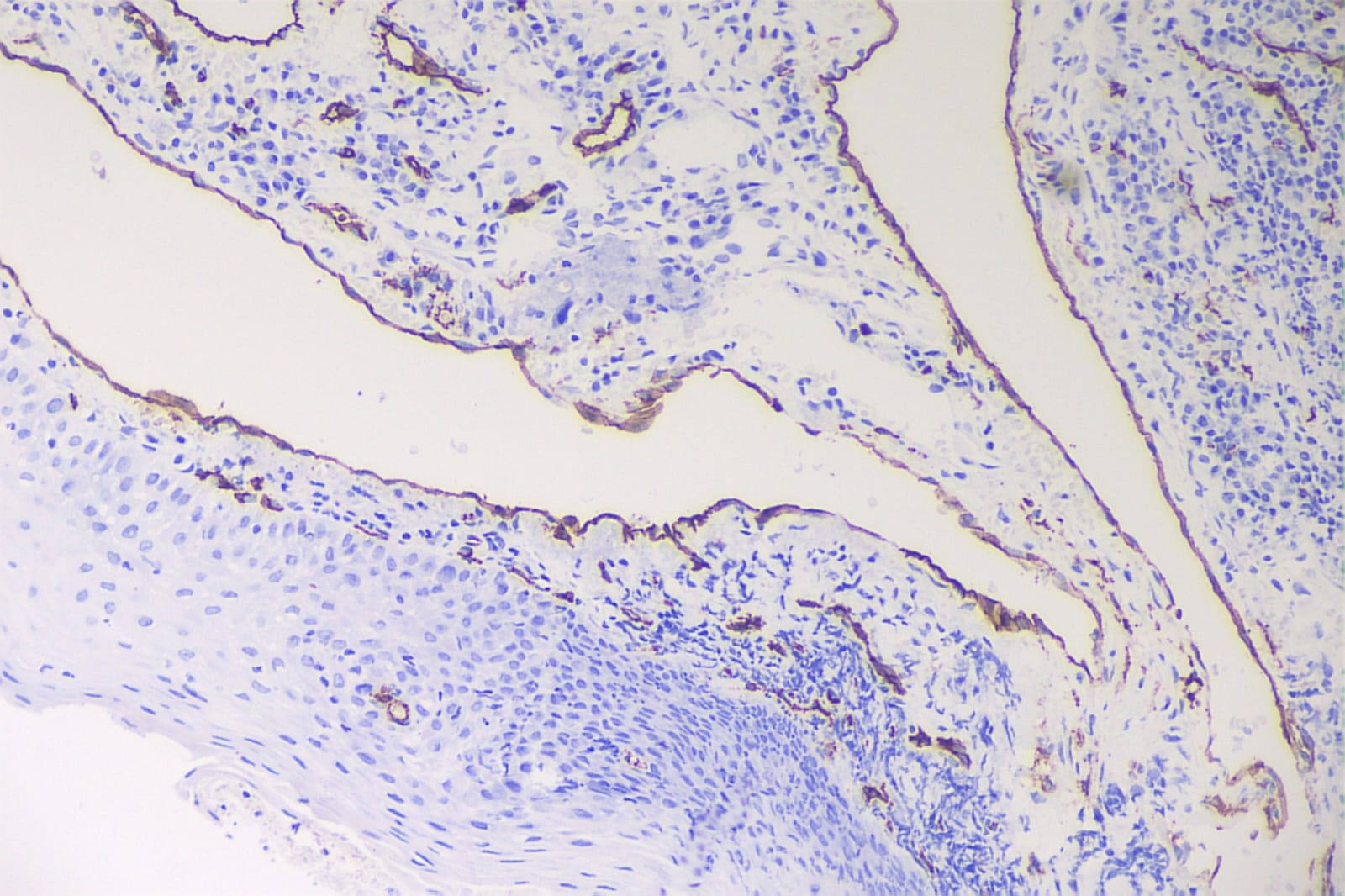

Supplement: Supplementary file 2 — Figure S2: Immunohistochemical staining for CD34 (magnification, 20×). [file LARY-136-794-s002.jpg]

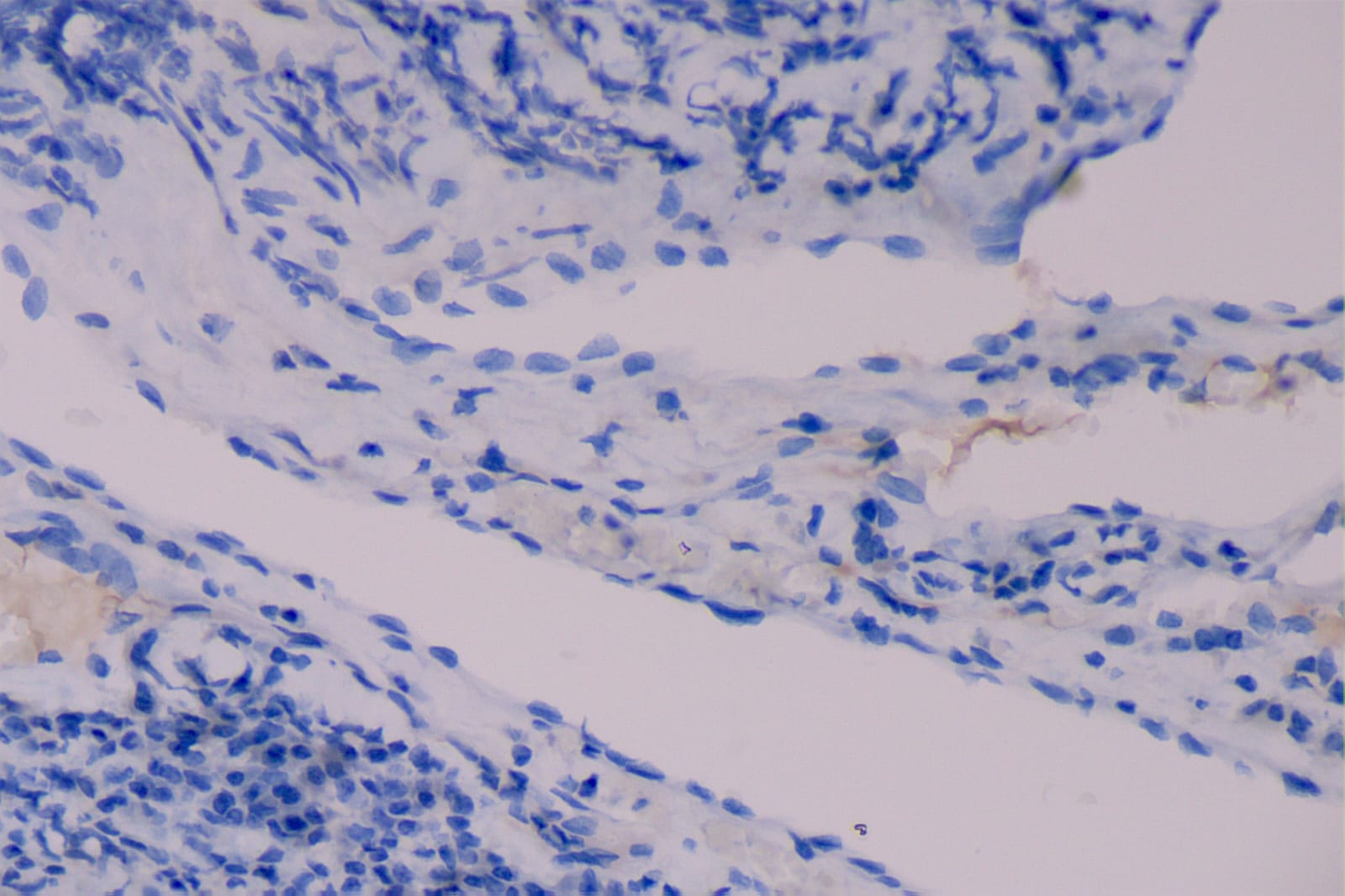

Supplement: Supplementary file 3 — Figure S3: Immunohistochemical staining for HHV8 (magnification, 40×). [file LARY-136-794-s004.jpg]
