# Supplementary material for: Rare Case of Vallecular Hemangioma With Complete Regression in an Adult Patient
Source: Laryngoscope. 2025 Sep 12;136(2):794–7. doi: 10.1002/lary.70128 (PMC12793954; doi:10.1002/lary.70128)
Supplement: Supplementary file 4 — Figure S4: Final pathology report confirming the diagnosis of vallecular hemangioma. [file LARY-136-794-s003.pdf]

LAUDO | IMUNO – HISTOQUIMICA

infolaudo laboratórios

EXAME: [redacted]

**Nome:** [redacted]	**Convênio:** CORTESIA
**Nascimento:** 02/09/1969	**Origem:** HOSPITAL UNIMED
**Sexo:** Masculino	**Destino:** HOSPITAL UNIMED
**Data de Coleta:** 20/08/2024	**Médico:** [redacted]
**Data de Entrada:** 07/07/2025	
**Data de Finalização:** 07/07/2025	

CONCLUSÃO

· **Valécula esquerda**

- HEMANGIOMA

Comentário: Trata-se de lesão vascular sem atipias, negativa para HHV8 e com mínimo índice proliferativo (Ki67).

Técnica utilizada

Todas as reações de imuno-histoquímica são realizadas de forma automatizada, nas plataformas Ventana (BenchMark ULTRA IHC/ISH System) ou Dako Agilent (Dako Autostainer), seguindo os protocolos dos fabricantes.

PAINEL DE ANTICORPOS

Anticorpo	Clone	Interpretação
CD34	QBEnd 10	Positivo nas células endoteliais
HHV-8	13B10	Negativo
Ki-67	MIB-1 (DAKO)	Baixo índice nas células endotelais (inferior a 1%)

IDENTIFICAÇÃO DA AMOSTRA

Exame realizado no bloco: 2200278991

infolaudo
laboratórios

EXAME: 0222006436007

Nome: ALESSANDRO LUIZ BARBERIO
Nascimento: 02/09/1969
Sexo: Masculino
Data de Coleta: 20/08/2024
Data de Entrada: 07/07/2025
Data de Finalização: 07/07/2025

Convênio: CORTESIA
Origem: HOSPITAL UNIMED
Destino: HOSPITAL UNIMED
Médico: MARCELO BELLI

Topografia	Cassete	Número	Lâmina	Mapeamento
Valécula esquerda	2200278991	2	CD34	Inclusão total
		3	Ki-67	
		4	HHV-8	

O presente laudo é uma análise interpretativa resultante da correlação de dados clínicos, laboratoriais e morfológicos. Os diagnósticos podem variar na dependência das informações contidas na requisição do exame, do emprego de técnicas especiais e da evolução dos conhecimentos científicos. Qualquer discordância frente ao laudo deverá ser imediatamente comunicada, postergando se medidas terapêuticas até que o caso seja revisado, pois a sensibilidade é a especificidade do método podem não ser absolutas, requerendo nova investigação.

Drª. Luciana Depiere Lanzarin
CREMESC 19520 RQE 14680
Médico Patologista Responsável
